# Gender-based variations in surgical management of colorectal liver metastases: comprehensive analysis

**DOI:** 10.1186/s12885-025-13612-3

**Published:** 2025-02-21

**Authors:** Pia F. Koch, Kristina Ludwig, Karl H. Hillebrandt, Hannes Freitag, Moritz Blank, Sebastian Knitter, Dominik Paul Modest, Felix Krenzien, Georg Lurje, Wenzel Schöning, Johann Pratschke, Igor M. Sauer, Simon Moosburner, Nathanael Raschzok

**Affiliations:** 1 Corporate Member of Freie Universität Berlin and Humboldt, Department of Surgery, Experimental Surgery, Charité– Universitätsmedizin Berlin, Universität zu Berlin, Campus Charité Mitte| Campus Virchow-Klinikum, Augustenburger Platz 1, 13353 Berlin, Germany; 2 Berlin Institute of Health at Charité– Universitätsmedizin Berlin, BIH Biomedical Innovation Academy, BIH Charité Clinician Scientist Program, Charitéplatz 1, 10117 Berlin, Germany

**Keywords:** Colorectal carcinoma, Gender, Colorectal liver-metastasis, Hepatic resection, Survival

## Abstract

**Background:**

Colorectal cancer with liver metastasis affects both men and women. However, therapeutic strategies and long-term outcomes could be influenced by patients’ sex, due to variations in tumour biology, lifestyle, and dietary habits. By conducting a comprehensive comparative analysis, this study aims to detail differences in tumour characteristics, postoperative complications, recurrence rates, and survival outcomes between sexes.

**Methods:**

Single-centre retrospective analysis between 2010 and 2022 of all patients undergoing liver surgery for colorectal liver metastases (CRLM) at the Department of Surgery, Charité– Universitätsmedizin Berlin. Patients were stratified by sex. Statistical analysis was performed using *R*V4.2.

**Results:**

We analysed 642 patients who underwent hepatic resections for CRLM. Baseline patient characteristics were comparable between sexes: However, significant differences (*p* < 0.001) were noted in body mass index (BMI), with females exhibiting lower BMIs (median BMI in females: 23.7 kg/m² vs. males: 26.5 kg/m²). Primary tumour locations varied significantly (*p* = 0.008), with females presenting more sigmoid colon tumours (37%), while males predominantly had rectal tumours (35%). RAS mutation rates were higher in females (54%) than males (34%, *p* = 0.005). A higher prevalence of bilobar metastases were evident in men (62%, *p* = 0.011), yet surgical techniques and complications showed comparable distributions. The time for resection was longer in males (median 304 min vs. 290 min in females); however, conversion to open surgery took place more often in females (5.2% vs. 2.3% in males). Postoperative complications and survival rates showed no significant differences by patients’ sex.

**Conclusion:**

Distinct sex-related patterns in tumour characteristics and postoperative outcomes in patients with CRLM were observed, emphasizing the need for further investigations to understand and address gender-based disparities for more personalized clinical management in the future.

**Trial registration:**

This research was conducted with ethical approval from the relevant institutional review board Ethikkommission der Charité– Universitätsmedizin Berlin’ (reference numbers EA2/006/16 and EA4/084/17). No other registration applied.

## Background

Colorectal cancer (CRC) is one of the most prevalent malignancies worldwide. Despite the availability of modern treatment options such as hepatic resection, radiofrequency ablation, chemotherapy, or immunotherapy [1], mortality rates remain high. Surgical resection of colorectal liver metastases (CRLM) is the only potentially curative therapy, offering 5-year survival rates of 25–58% [2–4]. Advances in surgical techniques, such as parenchymal-preserving resections and vascular R1 strategies, have significantly improved outcomes by ensuring adequate future liver function while maintaining excellent oncological results [5]. These factors influence the outcome in terms of recurrence-free-survival (RFS) and overall survival (OS) and are therefore of outmost relevance [2, 6]. However, emerging evidence across various fields of medicine indicate that there could be gender-related variations, influencing the pre-operative tumour characteristics as well as post-operative long-term outcomes with different result for male and female patients [7, 8], an area long overlooked due to historical biases in research, such as a preference for male subjects in preclinical studies [9]. In CRC, sex-related differences are well-documented. Male patients have historically exhibited a higher incidence of CRC, with an age-standardized rate of 20.0 per 100,000 men compared to 15.1 per 100,000 women in Western Europe in 2020 [10]. Recent trends suggest a narrowing of this gap between genders due to various factors such as lifestyle changes, dietary habits, and increased awareness leading to earlier detection in females [11, 12]. Additionally, women are more likely to present with right-sided tumors, which are associated with higher RAS mutation rates and often considered more aggressive [13, 14], Furthermore, several population-based studies highlighted that men are more often considered for surgical intervention [4, 15] while women had about 24.5% less of a chance to undergo liver surgery for CLRM [13, 16, 17]. There is a lack of records for long-term follow ups after metastasis resection regarding RFS and OS of patients according to their gender, although male patients have overall higher postoperative mortality [18, 19]. Understanding the influence of patients` sex on the outcomes of liver resection for colorectal liver metastasis could be crucial in advancing personalized medicine and refining treatment strategies. This research paper aims to analyse the nuanced relationship between patient sex and the outcomes of liver resection in the context of CRLM. By conducting a comprehensive comparative analysis, this study intends to highlight potential disparities in postoperative complications, recurrence rates, survival outcomes, and treatment responses based on the sex of the patient.

## Methods

### Study design

**Fig. 1:**
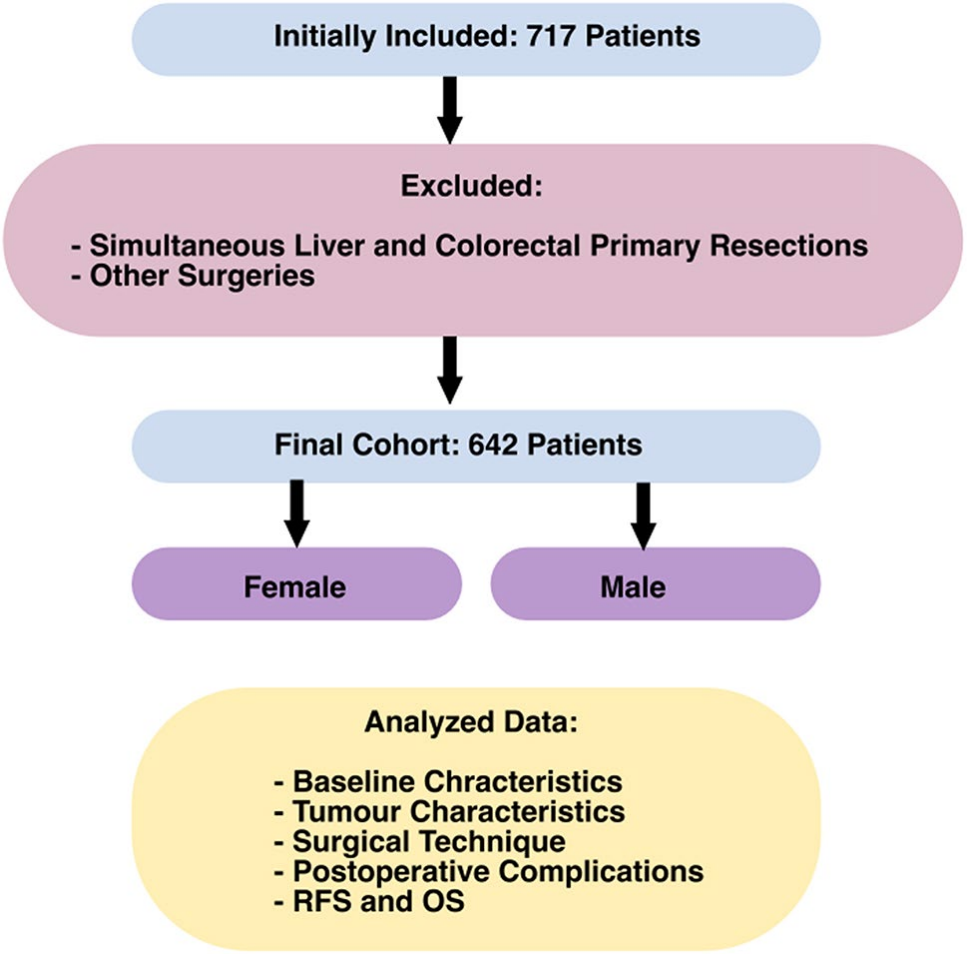
Flow chart illustrating the inclusion and exclusion of patients in the study and the analyzed data

This study is a single-centre retrospective analysis conducted at the Department of Surgery, Charité– Universitätsmedizin Berlin. The observational timeframe spanned from January 1st, 2010, to December 31st, 2022, with a focus on patients who underwent hepatic resection for CRLM. The primary aim was to stratify these patients by sex and analyse differences in clinical and pathological features. A total of 717 patients initially met the inclusion criteria, with the final analysis incorporating 642 patients. Patients who underwent simultaneous liver and colorectal primary resection or other surgeries were excluded from the study. The specific objectives aimed to compare the baseline characteristics and clinical features between male and female patients, investigate characteristics of the primary tumour, analyse CRLM quality variations, examine surgery techniques and corresponding postoperative outcomes, and evaluate both survival rates and complications (Fig. 1).

Baseline patient and tumour characteristics were extracted from the electronic health record in addition to extent of resection and postoperative data. Follow-up for overall and recurrence-free survival was provided by the Charité Comprehensive Cancer Centre.

### Statistical analysis

Statistical analysis was performed using *R* software, version 4.3.2 (R Foundation for Statistical Computing, Vienna, Austria). Packages for analysis and graph plotting were tidyverse, gtsummary, survminer and survival. The Mann-Whitney U test was utilized to evaluate differences between male and female patients in various clinical and pathological parameters. Kaplan-Meier survival curves were generated to compare survival outcomes, and the log-rank test was used to compare differences in survival rates between the two groups. Additional subset analyses were conducted to explore specific aspects of the data in more detail. A p-value < 0.05 was considered statistically significant.

## Results

### Patient characteristics and clinical features

642 patients were included in this study, all fulfilling the criteria of having colorectal liver metastases and receiving some form of hepatectomy, excluding simultaneous liver and colorectal primary resections. Patients were stratified by sex and differences between preoperative baseline characteristics and clinical aspects compared (Table 1), as well as differences in surgical approaches and postoperative outcome (Table 2) between males and females. The median age was almost identical between both groups. Females were in average 60 years of age, males 61 years, with a p-value of 0.7, indicating no significant difference. Body Mass Index (BMI) was significantly different (*p* < 0.001) between the two sexes, with females having a median BMI of 23.7 kg/m² and males 26.5 kg/m². The distribution of the American Society of Anaesthesiologists (ASA) score was similar across sexes (*p* = 0.9). There was also no distinct difference between the groups in the prevalence of alcohol abuse (5.2% in females vs. 9.1% in males, *p* = 0.087) and smoking (9.4% in females vs. 8.6% in males, *p* = 0.8).

**Table 1:** Preoperative tumour and patient characteristics

Variable	Overall, *n* = 642^*1*^	Female, *n* = 213^*1*^	Male, *n* = 429^*1*^	*p*-value^2^
**Age [years]**	61 (53, 70)	60 (51, 71)	61 (54, 69)	0.7
**ASA Score**				0.9
1	13 (2.2%)	3 (1.5%)	10 (2.5%)	
2	278 (47%)	91 (46%)	187 (47%)	
3	303 (51%)	103 (52%)	200 (50%)	
4	3 (0.5%)	1 (0.5%)	2 (0.5%)	
**BMI [kg/m** ^**2**^ **]**	25.6 (22.6, 29.1)	23.7 (21.2, 28.0)	26.5 (23.8, 29.4)	< 0.001
**History of alcohol abuse**	50 (7.8%)	11 (5.2%)	39 (9.1%)	0.087
**History of smoking**	57 (8.9%)	20 (9.4%)	37 (8.6%)	0.8
**Diabetes mellitus**	34 (5.3%)	8 (3.8%)	26 (6.1%)	0.3
**Cirrhosis**	12 (1.9%)	3 (1.4%)	9 (2.1%)	0.8
**Fibrosis**	101 (16%)	37 (17%)	64 (15%)	0.4
**Location of Primary Tumour**				0.008
Ascending colon	75 (12%)	26 (12%)	49 (11%)	
Cecum	30 (4.7%)	19 (8.9%)	11 (2.6%)	
Descending colon	42 (6.5%)	16 (7.5%)	26 (6.1%)	
Not specified	1 (0.2%)	1 (0.5%)	0 (0%)	
Rectum	240 (37%)	66 (31%)	174 (41%)	
Sigmoid colon	227 (35%)	78 (37%)	149 (35%)	
Transverse colon	27 (4.2%)	7 (3.3%)	20 (4.7%)	
**Location of Primary Tumour**				0.051
Left	509 (79%)	160 (75%)	349 (81%)	
Right	132 (21%)	52 (24%)	80 (19%)	
**RAS**				0.005
mutated	94 (41%)	43 (54%)	51 (34%)	
wildtype	137 (59%)	37 (46%)	100 (66%)	
**BRAF**				0.8
mutated	17 (11%)	5 (10%)	12 (12%)	
wildtype	131 (89%)	45 (90%)	86 (88%)	
**Time to metastasis**				0.7
Metachronous	281 (44%)	90 (43%)	191 (45%)	
Synchronous	352 (56%)	118 (57%)	234 (55%)	
**Bilobular metastases**	377 (59%)	110 (52%)	267 (62%)	0.011

^*1*^ Median (IQR); n (%)

^*2*^ Wilcoxon rank sum test; Fisher’s Exact Test for Count Data with simulated p-value (based on 2000 replicates); Fisher’s Exact Test for Count Data

**Table 2:** Surgical characteristics and postoperative outcome

Variable	Overall, *n* = 642^*1*^	Female, *n* = 213^*1*^	Male, *n* = 429^*1*^	*p*-value^2^
**Type of Surgery**				0.9
Laparoscopic	179 (28%)	62 (29%)	117 (28%)	
Open Surgery	412 (65%)	135 (64%)	277 (65%)	
Robotic assisted	44 (6.9%)	15 (7.1%)	29 (6.9%)	
**Extent of Liver Resection**				0.2
Extended left hepatectomy	16 (2.5%)	4 (1.9%)	12 (2.8%)	
Extended right hepatectomy	72 (11%)	21 (9.9%)	51 (12%)	
Left hepatectomy	69 (11%)	31 (15%)	38 (8.9%)	
Left lateral hepatectomy	19 (3.0%)	2 (0.9%)	17 (4.0%)	
Right hepatectomy	178 (28%)	58 (27%)	120 (28%)	
Right posterior sectionectomy	7 (1.1%)	3 (1.4%)	4 (0.9%)	
Segmentectomy	234 (36%)	79 (37%)	155 (36%)	
Wedge resection	47 (7.3%)	15 (7.0%)	32 (7.5%)	
**Conversion to Open Surgery**	21 (3.3%)	11 (5.2%)	10 (2.3%)	0.063
**Surgery Duration [min]**	300 (247, 370)	290 (241, 361)	304 (251, 373)	0.12
**Length of Intensive Care Unit Stay [days]**	1.00 (1.00, 2.00)	1.00 (1.00, 2.00)	1.00 (1.00, 2.00)	0.4
**Length of Hospital Stay [days]**	10 (8, 16)	10 (8, 15)	11 (8, 16)	0.14
**Clavien-Dindo**				> 0.9
1	46 (7.6%)	15 (7.3%)	31 (7.8%)	
2	35 (5.8%)	14 (6.8%)	21 (5.3%)	
3a	43 (7.1%)	14 (6.8%)	29 (7.3%)	
3b	34 (5.6%)	11 (5.4%)	23 (5.8%)	
4a	8 (1.3%)	1 (0.5%)	7 (1.8%)	
4b	4 (0.7%)	2 (1.0%)	2 (0.5%)	
5	14 (2.3%)	5 (2.4%)	9 (2.3%)	
**Time between Diagnosis and Liver Surgery [months]**	11 (6, 25)	10 (5, 23)	12 (6, 27)	0.14

^*1*^ Median (IQR); n (%)

^*2*^ Wilcoxon rank sum test; Fisher’s Exact Test for Count Data with simulated p-value (based on 2000 replicates); Fisher’s Exact Test for Count Data

For clinical features, the incidence of diabetes mellitus (3.8% in females vs. 6.1% in males, *p* = 0.3) and cirrhosis (1.4% in females vs. 2.1% in males, *p* = 0.8) were compared between groups.

Additionally, fibrosis of the liver was investigated in all patients: 17% of females and 15% of males had histopathologically confirmed fibrosis (*p* = 0.4).

### Primary tumour characteristics

A difference was found in the location of the primary tumour between groups (*p* = 0.008): Female patients had mostly occurrences of sigmoid colon tumours (*n* = 78, 37%), while males had predominately primary rectum tumours (*n* = 149, 35%). Both sexes had the least occurrences in transverse colon tumours (females: 3.3%; males: 4.7%).

Moreover, tumour histology was examined regarding RAS and BRAF mutation status, highlighting a significant difference between patient’s sex: RAS mutation was detected in 54% of analysed female samples and only in 34% in male ones (*p* = 0.005). BRAF mutation rates however, showed no significant distributions (10% in females vs. 12% in males, *p* = 0.8).

### Liver metastases

Furthermore, differences in CRLM were analysed between groups. No significant distinction was visible in the time to metastases (time in days between first cancer diagnosis and occurrence of CRLM) across sexes (*p* = 0.7). Female and male patients both had more often synchronous (females: *n* = 118, 57%), males: *n* = 234, 55%) than metachronous metastases (females: *n* = 90, 43%), males: *n* = 191, 45%), (*p* = 0.7). A significant difference was detected in the dissemination of metastasis within the liver, revealing that more men (62%) had bilobular metastases than women (52%), with *p* = 0.011.

### Surgery technique and postoperative outcome

Types of surgery, containing laparoscopic, open, and robotic procedures were similar across sexes (*p* = 0.9). Conversion to open surgery when started with a minimal-invasive technique was necessary more often in women (5,2%) than men (2,3%), only slightly missing a statistical significance (*p* = 0.063). Median duration of surgery was moderately longer in males (304 min) compared to females (290 min), although without a significant difference (*p* = 0.12), time between diagnosis of CRLM and liver surgery was similar in both sexes as well (women 10 months; men: 12 months).

Results of the extent of liver resection showed differences in numbers and percentages between sexes but without statistical significance (*p* = 0.2): More than twice as many men obtained right hepatectomies, compares to women in absolute numbers (*n* = 120 male vs. 58 female). Concomitant, male patients received extended right hepatectomies more often (12%) and left lateral hepatectomies (4.0%) than female patients (9.9% and 0.9%, respectively). Another significant distinction was detectable in postoperative bilirubin levels al various time points, with higher levels in males (*p* < 0.001 for POD 1 and for POD 5; *p* = 0.019 for final levels). Furthermore, the international normalized ratio (INR) showed significant differences at POD 5 and in the final measurement, with higher values in males (*p* = 0.003 and *p* < 0.001, respectively).

### Postoperative morbidity and mortality

**Fig. 2:**
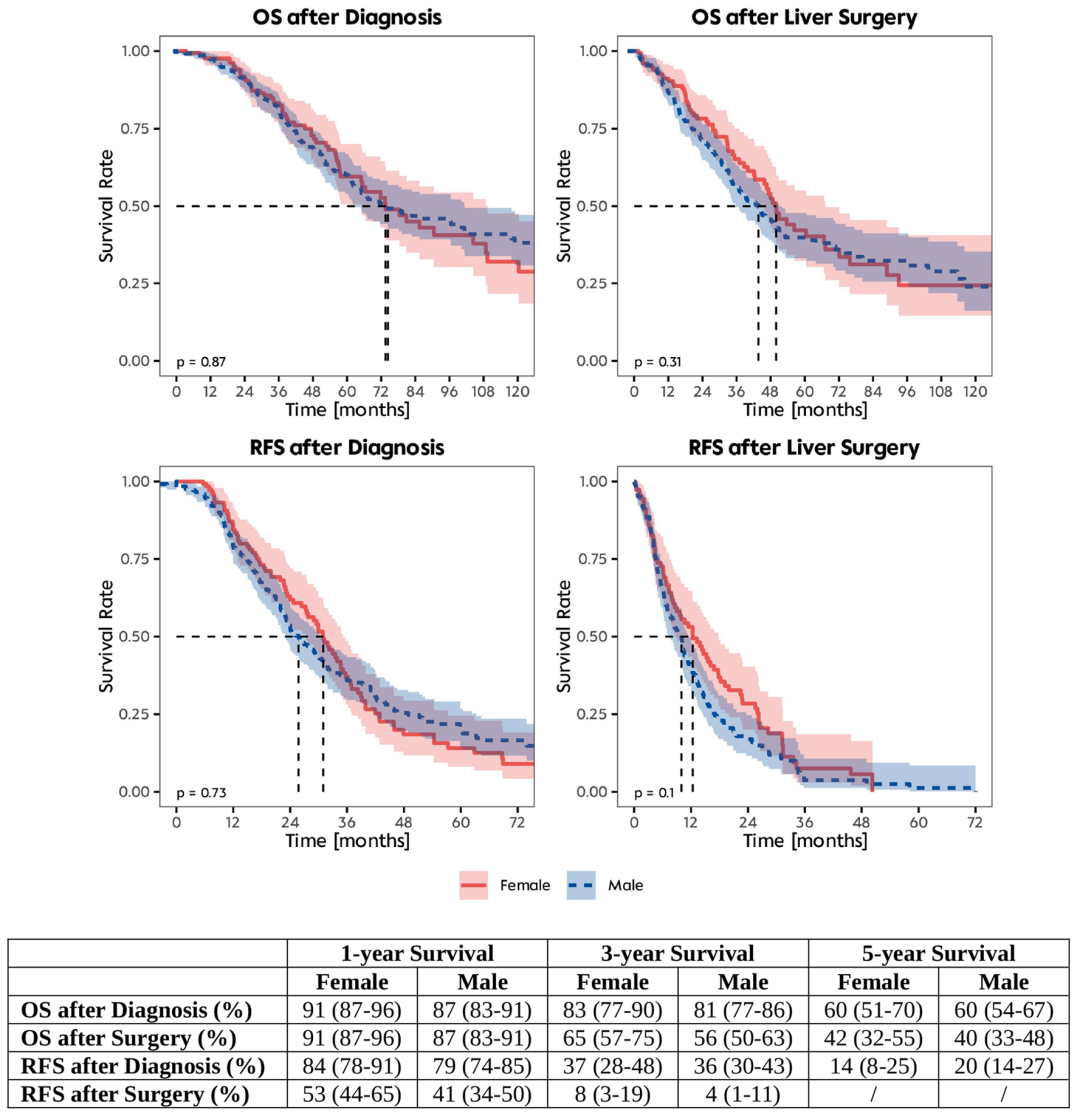
Kaplan-Meier survival curves. OS: Overall survival; RFS: Recurrence-free survival; Dotted black lines: Median Survival. Survival Curves were compared using the log-rank test

Finally, postoperative complications were compared between females and males and recurrence-free survival and overall-survival rates were calculated for both groups. Women and men had comparable time periods of overall length of hospital stay and ICU stay (*p* = 0.14 for hospital stay; *p* = 0.4 for ICU stay). The distribution of postoperative complications according to the Clavien-Dindo classification was similar across sexes (*p* > 0.9). OS and RFS rates are shown after diagnosis and after surgery in female and male patients (Fig. 2). Both sexes exhibit high one-year survival rates after diagnosis, with females slightly outperforming males in both OS and RFS (female: 91% OS, 84% RFS; male: 87% OS, 79% RFS). The three-year survival rates decline notably compared to one-year rates for both sexes. Again, both sexes show a considerable drop in survival rates by the fifth-year post-diagnosis, with females having a similar OS but slightly lower RFS in the median (60% OS, 14% RFS) compared to males (60% OS, 20% RFS). After surgery, all patients demonstrated high one-year survival rates (females: 91%; males: 87%), but men generally showed lower RFS compared to females after one year (females: 53%; males: 41%) and after three years (females: 8%; males: 4%). OS rates dropped three years after surgery for both sexes (females: 65%; males: 56%) and again after five years (females: 42%; males: 40%), demonstrating no notable difference in long-term outcome according to patients’ gender.

## Discussion

The present study, encompassing a cohort of 642 patients undergoing liver surgery for colorectal liver metastases (CRLM), provides critical insights into the interplay among patient characteristics, clinical features, outcomes post-surgery and gender. Notably, the median age and distribution of American Society of Anaesthesiologists (ASA) scores were remarkably similar between genders, suggesting a comparable baseline health status. However, a notable difference in BMI was observed, with males exhibiting a higher median BMI compared to their female counterparts.

The observation that men have higher BMIs than women is in line with modern trends in German adults and can be attributed to several factors [20, 21]. Men and women have different biological and hormonal compositions, which can affect fat distribution and muscle mass. Testosterone, more prevalent in men, promotes muscle growth and can influence the distribution of body fat [22]. However, as men age, they may experience a decrease in testosterone levels, which can lead to increased fat accumulation, particularly around the abdomen [23]. Recent literature suggests a connection between abdominal fat, adipokines, aging and possibly gender [24]. In addition to these factors, studies have demonstrated that postprandial signals varied between men and women, influencing the BMI [25].

Concerning the primary tumours, male patients presented with a much larger share in rectum tumours than females. These findings are in line with the current data: In 2020, 443,358 male patients were diagnosed with rectal cancer, with an age-standardized-rate of 9.8 per 100,000 person-years, whereas only 288,852 female patients received this diagnosis with an age-standardized-rate of 5.6 per 100,000 person-years worldwide [10]. The interplay of genetics, lifestyle, hormonal influences, and environmental factors might collectively influence tumour development in the rectum and other parts of the colon [26]. For instance, women have higher oestrogen levels, which can have protective effects against tumours in certain areas [27]: Modern studies pointed out the role of circulating oestrogen as a tumour suppressor, not only in breast cancer, but in colorectal cancer as well [28, 29]. Variances in dietary habits and lifestyle factors might also contribute: Men tend to consume diets higher in red meat and lower in fibre, both of which have been associated with increased risks of colorectal cancer, particularly in the rectum [30].

We observed higher RAS mutation rates in women. Research on differences in RAS mutation distribution between male and female patients is limited, but a survey of pathology centers across Europe observed that the prevalence of RAS mutations was associated with the location of the primary tumour: Higher mutation rates were found in right-sided than left‐sided tumors (54.6% vs. 46.4%, respectively) [31] Women present more often right-sided primary tumours [13, 32], and thus a higher prevalence of RAS mutation. Patients with RAS mutations have been described to suffer from a more aggressive course of disease, including faster tumour growth, higher likelihood of metastasis and poorer overall survival and disease-free survival rates [33], due to resistance to specific targeted therapies (anti-Epidermal Growth Factor Receptor therapies) [34, 35]. One potential factor could again be different oestrogen influences in women and men, as oestrogen receptors can impact various signalling pathways, potentially affecting the likelihood of RAS mutations [36]. Similar pathways regarding oestrogen could also contribute to the higher prevalence of bilobular metastases in males, restricting or altering the dissemination of such in females. These mechanisms regarding oestrogen were already seen in breast cancer studies, where oestrogen signalling had a protective effect in later stages, while oestrogen receptor loss was associated with an aggressive metastatic disease [37]. Overall, this higher prevalence of RAS mutations in females could influence the selection and timing of neoadjuvant or adjuvant treatments when investigated further.

Other explanations for distribution of metastases within the liver may be attributed to variances in liver anatomy between men and women [38–40].

Recent records claim that a complex microenvironment in the liver plays a significant role to metastatic dissemination [41]. This microenvironment consists of cells, extracellular matrix, as well as recruited inflammatory and immune cells, which all participate in the response to invading tumour cells and could inhibit or favour the progression of metastasis [42]. It has already been reported that there are gender differences in immune cells and immune responses [43], which could explain varied microenvironments and therefore divergent metastasis patterns in men and women.

Both sexes displayed similarities in time to metastases, time between diagnosis and surgery, and the surgical technique employed, suggesting comparable disease progression and surgical approaches between male and female patients. However, the differences in the dissemination of liver metastases (bilobular metastases being more common in males, 62% vs. 52%, *p* = 0.011) could affect surgical planning, with male patients benefiting from more aggressive surgical or ablative approaches to address the complexity of bilobular disease, while female patients with unilobar disease might be candidates for more conservative resections, preserving liver function.

While not reaching statistical significance, there were higher instances of conversion from minimal invasive procedures (laparoscopic or robotic assisted) to open surgery observed in female patients compared to male patients. This observation might be caused by a more complex anatomical situation in female patients, possibly due to a higher likelihood of prior abdominal surgeries and consequently intrabdominal adhesions in women or even hint at physiological factors beyond body habitus, potentially related to variations in tissue elasticity or vascularity. Most literature states that obesity is one of the most important risk factors for conversion to open surgery [44]. In our cohort, however, women had lower BMIs. The significantly higher median BMI in males likely contributes to certain technical challenges during surgery, potentially explaining the longer median operative duration in males (304 min vs. 290 min, *p* = 0.12) and the differences in the extent of liver resections performed.

Similar outcomes were observed in both patient groups concerning postoperative complications and long-term survival rates. Immediately following the diagnosis of CRLM and subsequent hepatic resection, OS and RFS were high in both sexes, gradually declining over time. Notably, men exhibited moderately inferior RFS rates, indicating a decrease in survival probability over time [45, 46].

However, notable differences were evident in postoperative bilirubin levels between women and men. This might be attributed to the higher number in extended resections (in men) and therefore impaired hepatic function after surgery with the result of elevated levels of bilirubin [47]. It could also be based on a combination of sex-related variations in liver function, recovery, and response to surgical stress, as seen in other trials [21, 48, 49]. Another explanation could arise from a complex interplay of physiological, anatomical, and biological factors unique to each sex, impacting bilirubin metabolism and clearance pathways. It is possible that patients had diverse baseline bilirubin levels upon admission, as previously reported in studies [50], which were not documented in this study.

This underscores the importance of establishing sex-specific reference levels, which would allow for more effective screening and personalized therapy monitoring [51]. A comprehensive understanding of distinct differences in disease characteristics between males and females holds the promise of enhancing overall patient care.

The study’s major strength is the high patient number and the extent of available characteristics for individual patients. However, it is important to note that the analysis relied on a retrospective single center study, which may introduce inherent selection and reporting biases. While the study controlled for several variables, residual confounding factors may still influence the results.

Additionally, the findings may not be generalizable to other populations due to differences in healthcare systems, surgical expertise, and patient demographics.

Despite these limitations, our findings could serve as a foundation for developing sex-specific preoperative risk assessments, optimizing surgical techniques, and refining postoperative care pathways, ultimately enhancing individualized treatment plans. Future studies with larger, well represented cohorts, specific focus on sex-based analyses, and interdisciplinary collaboration could help fill this research gap and provide a more comprehensive understanding of the molecular intricacies in patients with CRLM undergoing hepatic surgery [52].

## Conclusion

Understanding and addressing gender-based disparities in patients with CRLM holds significant clinical importance and could leading to the development of tailored therapeutic strategies that optimize treatment efficacy and postoperative care [51, 53]. Promoting dedicated sex-related research is pivotal for enabling personalized management of CRLM, ensuring optimized therapeutic approaches and better long-term outcomes for both male and female patients.

## Data Availability

The datasets generated and/or analysed during the current study are not publicly available due data protection laws in Germany but are available from the corresponding author on reasonable request. Data are located in controlled access data storage at Charité– Universitätsmedizin Berlin.

## Abbreviations

ASA: American Society of Anaesthesiologists
BMI: Body Mass Index
CI: Confidence interval
CRC: Colorectal cancer
CRLM: Coloretal liver metastasis
OS: Overall survival
POD: Post-operative day
RFS: Recurrence-free-survival
